# Photobiology and Endogenous Fluorophore Based Applications, from Natural Environment to Biomedicine to Improve Human Life

**DOI:** 10.3390/molecules25235707

**Published:** 2020-12-03

**Authors:** Anna C. Croce

**Affiliations:** 1 Institute of Molecular Genetics, Italian National Research Council (CNR), Via Abbiategrasso 207, I-27100 Pavia, Italy; croce@igm.cnr.it; Tel.: +39-0382-986-428; 2 Department of Biology & Biotechnology, University of Pavia, Via Ferrata 9, I-27100 Pavia, Italy

Light interaction with biological molecules is commonly acknowledged to depend on the optical properties of these compounds and may result in the activation of events with various beneficial or harmful outcomes in living organisms. These issues are encompassed in the photobiology science, together with unceasing and extensive studies intended to advance the knowledge on light interaction with biological systems, and to develop applications to improve environment and human life. The journal *Molecules* and its Photochemistry Section are greatly contributing to a continuous comprehensive update in the panorama of photobiology and naturally fluorescing biomolecules, as demonstrated by the numerous papers recently published therein, some of which are recalled in this Editorial.

The sun is the unique, natural energy source, which has directed the development of biomolecules with absorption properties suitable to capture solar light and convert it into chemical energy, essential for the growing and evolution of living organisms. Chlorophylls and carotenoid are the pigments contained in plant photosynthetic antennae engaged in the harvesting of light energy and its transfer, making algae and plants the primary producers of biomass in the flow of maintenance of the whole biosphere. The photophysical properties of chlorophylls have been widely investigated since a long time, from the basic characterization of their absorption and fluorescence spectral properties to the energy transfer and exciton events. Anyway, some intriguing aspects are continuously newly revealed. This is the case of the near-infrared spectral region of chlorophyll α, for which a very recent characterization by means of anti-Stokes fluorescence excitation spectroscopy has shown the novelty of a weak, although reliably detectable, band in the red absorption tail. Because of the spectral position and weak absorption, this band relatable to intramolecular vibrational modes has been suggested unlikely to contribute directly to light harvesting, while a possible role could be played in energy transfer events in the network of photosynthetic antennae [1]. Carotenoids, in addition to participating in light harvesting in photosystems, interact with chlorophylls in the regulation of photosynthesis energy flow. A spectral study on chlorophyll α and β-carotenoid as pure compounds in a solution confirmed that the two pigments may interact with two mechanisms, electron transfer and energy transfer events, the balance of which is affected by the polarity of the solvent. As a consequence, caution is recommended in the choice and use of solvents when studying photosystems in solutions [2]. Moreover, the derivatives of carotenoids containing oxygen, namely xanthophylls, are essential in plant photoprotection strategies because of their involvement in both non-photochemical quenching (NPhQ) and scavenging of reactive oxygen species (ROS). An NPhQ photoprotection mechanism commonly operating in plants and algae is based on the cycle conversion of de-epoxidized xanthophyll molecules, as recalled in a review on 9’-cis-neoxanthin and its participation in photoprotection, and its likely but still not completely elucidated role in photoisomerization and ROS scavenging [3]. Antioxidant protection by carotenoids has evolved in plants and has translated to animals and humans, in turn sharing with plants part of ROS generating causes. In general, animals and humans cannot synthetize carotenoids, while they are able to synthetize their retinoid derivatives. Antioxidants mechanisms in plants and humans are based on Zeaxanthin and lutein, respectively, the derivative of β-carotene involved the xantholphyll cycle and the structural isomer derived from α carotene, which have been the subject of a review paper, which received great attention from the scientific community, to the extent of becoming part of the Top Downloaded Articles in July–October 2020 [4]. This review underscores the concept that in both plants and animals the modulation of ROS production by antioxidants may result in both beneficial and hazardous effects, in a balance dependent on the pre-existing biometabolic and environmental context. In this view, a diet supplementation with carotenoid derivatives and other antioxidants aiming to improve healthy life has been suggested to encompass a careful analysis of the redox state of the subject and of the factors influencing it, such as life style, metabolic disorders or pathologies.

For a long time, photoxidation events induced by the light irradiation of photosensitizing molecules have been investigated for various beneficial applications. The massive and unceasing efforts devoted to the advance of photodynamic diagnosis and therapy strategies for the treatment of cancer and other pathologies, and for microbial inactivation, based on the development of increasingly efficient photosensitizers from early natural compounds [5,6,7], are beyond the aim of this Editorial. Nevertheless, it is worthwhile to recall the particular case of the impact on water organic substances from photochemical processes dependent on inorganic compounds, halide ions and halogens, such as chloride and bromide. Non-radical and radical reactive species in natural waters may be derived from these compounds following the UV-activated photolysis or reactions with oxidizing species formerly produced by natural solar light, such as ozone, hydroxyl and sulfate radicals, and in general ROS. Halogens and halide ions in photochemical processes and photoinduced halogenation reactions may have complex effects on the natural environment, the natural cycling of halogens and the decomposition of organic matter in terrestrial and oceanic water, and on the treatment of pollution and wastewaters. Attention is anyway to be paid also to undesired production of toxic derivatives [8].

As for the diagnostic applications in biomedicine based in the direct detection of endogenous fluorophores, we must remember that endogenous fluorophores in living organisms are powerful intrinsic biomarkers, due to their ubiquitous presence, involvement in metabolic and catabolic reactions and in tissue architecture, and their changes occurring under physiological conditions or following the rising of disorders and pathologies. An example is given by the intracellular accumulation of the heterogeneous products of oxidation of lipids, nucleic acids and proteins, which have been proposed to contribute to cell fluorescence in the red and near infrared regions. Despite this signal is a possible cause of disturbance in imaging analysis based on exogenous fluorophores as labeling agents, these red fluorescing oxidation products could play a role as an additional diagnostic endogenous biomarker of oxidative stress or of particular cell and tissue bio-metabolic conditions in autofluorescence-based investigations [9]. In this respect, such a kind of fluorophores might at least partially explain the near infrared fluorescence of parathyroid, currently exploited in the clinics to support the intraoperative discrimination of these glands during the surgical operation of thyroid or parathyroid [10]. These two latter reports have contributed to the Special Issue “Autofluorescence Spectroscopy and Imaging”, aimed to promote the knowledge on autofluorescence analysis for manifold diagnostic applications. Going back to chlorophyll, its autofluorescence has been proposed as a diagnostic biomarker to monitor the growing and quality of microalgae, with the perspective of applications on environmental surveillance, aquaculture, production of biomass as well as of food and chemical compounds to be used in the health and medical fields [11]. Plants, besides chlorophylls and its fluorescence as an important biomarker for the surveillance of their physiological conditions also via remote systems, several additional fluorophores can be exploited for very different purposes. An example is lignin, useful for the phenotyping or assessing the chemical modification of wood. Valuable results have also been obtained by fluorescence induction following aldehyde fixation of pine needles for the imaging diagnosis of fungal infestation [12]. As for the clinics, a systematic revision has recalled various application based on autofluorescence analysis as a common diagnostic tool to detect neoplastic lesions in the oral cavity, gastrointestinal tract, bronchial mucosa, hidden caries in dentistry, or to assess different diseases such as skin damage, the skin accumulation of advanced glycation products as biomarkers of diabetes or kidney and/or cardiovascular disfunction, lung ischemia and the risk of rejection after transplantation, and again, as a help in the preservation of parathyroid during surgical operation [13]. These reports have been incisively considered, leading to the conclusion that autofluorescence can generally help diagnosis, in some case more to inexperienced than experienced operators, and that in any case a still lacking standardization of operative and analysis procedures is recommended, to improve the real time, cost effective application of autofluorescence in the clinical diagnosis. These suggestions can be valid also to advance the autofluorescence-based diagnosis of liver metabolic disorders and disease progression, for which promising results have been reported from animal models, to assess and monitor the risk of progression to severe pathology [14,15,16]. Beyond the identification of endogenous fluorophores as biomarkers of the metabolic and morphological condition of a biological substrate in which they are directly involved, it is to underscore the conceptual advance given by the promising attempt to put autofluorescence in relation with the status of genes, such as p53 and p16, regulating cell proliferation and their consequent engagement in cell metabolism [17].

The intrinsic spectral properties of peptides and proteins are also at the basis of multipurpose investigations by means of spectroscopic techniques. A particular application concerns the control of the freshness of seafood and of the effects of freezing and thawing, which affect the quality and texture of the muscle by inducing the physical damages, autolysis, degradation and oxidation of proteins and lipids. To detect these changes in the absorption and fluorescence spectroscopy of the overall near UV–visible-red and near infrared range are increasingly considered cost effective, sensitive and real time techniques for the quality control of seafood [18].

In conclusion, beyond fields which have been overlooked in this editorial, such as specific photochemical studies on chemically synthesized organic and inorganic molecules and exogenous dyes used as fluorescing labeling agents and phototherapy, photobiology and autofluorescence-based issues are extensively represented by the various effective papers timely published in *Molecules*.

## References

[1] Leiger K., Linnanto J.M., Freiberg A. (2020). Establishment of the Qy Absorption Spectrum of Chlorophyll a Extending to Near-Infrared. Molecules.

[2] Chen C., Gong N., Li Z., Sun C., Men Z. (2017). Concentration effect on quenching of chlorophyll a fluorescence by all-trans-β-carotene in photosynthesis. Molecules.

[3] Giossi C., Cartaxana P., Cruz S. (2020). Photoprotective role of neoxanthin in plants and algae. Molecules.

[4] Demmig-Adams B., López-Pozo M., Stewart J.J., Adams W.W. (2020). Zeaxanthin and lutein: Photoprotectors, anti-inflammatories, and brain food. Molecules.

[5] Kessel D. (2019). Photodynamic Therapy: A Brief History. J. Clin. Med..

[6] Kessel D., Oleinick N.L. (2018). Cell Death Pathways Associated with Photodynamic Therapy: An Update. Photochem. Photobiol..

[7] Mesquita M.Q., Dias C.J., Neves M.G.P.M.S., Almeida A., Faustino M.A.F. (2018). Revisiting current photoactive materials for antimicrobial photodynamic therapy. Molecules.

[8] Yang Y., Pignatello J.J. (2017). Participation of the halogens in photochemical reactions in natural and treated waters. Molecules.

[9] Semenov A.N., Yakimov B.P., Rubekina A.A., Gorin D.A., Drachev V.P., Zarubin M.P., Velikanov A.N., Lademann J., Fadeev V.V., Priezzhev A.V. (2020). The oxidation-induced autofluorescence hypothesis: Red edge excitation and implications for metabolic imaging. Molecules.

[10] Ladurner R., Lerchenberger M., al Arabi N., Gallwas J.K.S., Stepp H., Hallfeldt K.K.J. (2019). Parathyroid autofluorescence—How does it affect parathyroid and thyroid surgery? A 5 year experience. Molecules.

[11] Takahashi T. (2019). Routine management of microalgae using autofluorescence from chlorophyll. Molecules.

[12] Donaldson L. (2020). Autofluorescence in plants. Molecules.

[13] Wizenty J., Schumann T., Theil D., Stockmann M., Pratschke J., Tacke F., Aigner F., Wuensch T. (2020). Recent advances and the potential for clinical use of autofluorescence detection of extra-ophthalmic tissues. Molecules.

[14] Croce A.C., Ferrigno A., Berardo C., Bottiroli G., Vairetti M., di Pasqua L.G. (2020). Spectrofluorometric analysis of autofluorescing components of crude serum from a rat liver model of ischemia and reperfusion. Molecules.

[15] Valor A., Arista Romeu E.J., Escobedo G., Campos-Espinosa A., Romero-Bello I.I., Moreno-González J., Fabila Bustos D.A., Stolik S., de La Rosa Vázquez J.M., Guzmán C. (2019). Study of methionine choline deficient diet-induced steatosis in mice using endogenous fluorescence spectroscopy. Molecules.

[16] Croce A.C., Ferrigno A., Bottiroli G., Vairetti M. (2018). Autofluorescence-based optical biopsy: An effective diagnostic tool in hepatology. Liver Int..

[17] Moriichi K., Fujiya M., Kobayashi Y., Murakami Y., Iwama T., Kunogi T., Sasaki T., Ijiri M., Takahashi K., Tanaka K. (2019). Autofluorescence imaging reflects the nuclear enlargement of tumor cells as well as the cell proliferation ability and aberrant status of the p53, Ki-67, and p16 genes in colon neoplasms. Molecules.

[18] Hassoun A., Shumilina E., di Donato F., Foschi M., Simal-Gandara J., Biancolillo A. (2020). Emerging techniques for differentiation of fresh and frozen-thawed seafoods: Highlighting the potential of spectroscopic techniques. Molecules.

